# Optimization of ʟ-ornithine production in recombinant *Corynebacterium glutamicum* S9114 by *cg3035* overexpression and manipulating the central metabolic pathway

**DOI:** 10.1186/s12934-018-0940-9

**Published:** 2018-06-13

**Authors:** Bin Zhang, Miao Yu, Wen-Ping Wei, Bang-Ce Ye

**Affiliations:** 1 Laboratory of Biosystems and Microanalysis, State Key Laboratory of Bioreactor Engineering, East China University of Science and Technology, Shanghai, 200237 China; 2 Collaborative Innovation Center of Yangtze River Delta Region Green Pharmaceuticals, College of Pharmaceutical Sciences, Zhejiang University of Technology, Hangzhou, 310014 Zhejiang China

**Keywords:** *Corynebacterium glutamicum*, ʟ-Ornithine, Metabolic engineering

## Abstract

**Background:**

ʟ-Ornithine is an important amino acid with broad applications in pharmaceutical and food industries. Despite lagging ʟ-ornithine productivity and cost reduction, microbial fermentation is a promising route for sustainable ʟ-ornithine production and thus development of robust microbial strains with high stability and productivity is essential.

**Results:**

Previously, we systematically developed a new strain, SO1 originate from *Corynebacterium glutamicum* S9114, for ʟ-ornithine production. In this work, overexpression of *cg3035* encoding *N*-acetylglutamate synthase (NAGS) using a plasmid or by inserting a strong P_*tac*_ promoter into the chromosome was found to increase ʟ-ornithine production in the engineered *C. glutamicum* SO1. The genome-based *cg3035* modulated strain was further engineered by attenuating the expression of *pta* and *cat*, inserting a strong P_*eftu*_ promoter in the upstream region of glycolytic enzymes such as *pfkA*, *gap*, and *pyk*, and redirecting carbon flux to the pentose phosphate pathway. The final strain with all the exploratory metabolic engineering manipulations produced 32.3 g/L of ʟ-ornithine, a yield of 0.395 g ornithine per g glucose, which was 35.7% higher than that produced by the original strain (23.8 g/L).

**Conclusion:**

These results clearly demonstrated that enhancing the expression of NAGS promoted ʟ-ornithine production and provide a promising alternative systematic blueprint for developing ʟ-ornithine-producing *C. glutamicum* strains.

**Electronic supplementary material:**

The online version of this article (10.1186/s12934-018-0940-9) contains supplementary material, which is available to authorized users.

## Background

Chemicals produced by metabolically engineered strains have gained major importance in industrial biotechnology, which is developing as a stimulating field due to the advantages of providing environmentally friendly products to replace petrochemicals [1]. With the development of gene manipulation tools, there is an increasing number of strains such as *Escherichia coli*, *Corynebacterium glutamicum*, *Bacillus subtilis* and others that have been modified to produce economically valuable products. *C. glutamicum* is a favored industrial microorganism with promising applications in the industrial production of amino acids such as ʟ-glutamate and ʟ-lysine at a million-ton scale in the past decades [2, 3]. Recently, increasing studies have used *C. glutamicum* for producing several products including diamines [4, 5], terpene [6], diols [7], and amino acids such as ʟ-isoleucine, ʟ-arginine, and ʟ-ornithine [3]. ʟ-Ornithine is an intermediate metabolite in the urea cycle, a key precursor for the biosynthesis of ʟ-citrulline, ʟ-proline, and polyamines, and a non-essential amino acid that plays a critical role in post-traumatic treatment, liver protection [8] and treatment of liver disease, strengthening the heart, and maintaining the working of the immune system [9].

Due to the importance of ʟ-ornithine in human health-promoting activities, economical and efficient production of ornithine has received much attention in the past years. The major strategy for producing ʟ-ornithine is focused on the enzymatic action of arginase on arginine and microbial fermentation. Metabolic engineering of microorganisms to produce ʟ-ornithine is an attractive alternative due to the high economic cost pressures of using ʟ-arginine for the enzymatic reaction. Recently, several reports have focused on the development of metabolically engineered strains that rapidly convert high concentrations of simple sugars to ʟ-ornithine. Jensen et al. constructed a metabolically engineered *C. glutamicum* strain that could produce 0.524 g ʟ-ornithine per g glucose in CgXII medium through disruption of *argFRG* and overexpression of *gdh* and *argCJBD* [10]. Hwang and Cho modulated the NADPH supply to ʟ-ornithine biosynthesis by inactivating three putative glucose dehydrogenases, which improved the yield of ʟ-ornithine up to 14 g/L [11]. Kim et al. developed a high ʟ-ornithine producing *C. glutamicum* strain by disrupting *argF*, *argR,* and *proB*, and overexpressing the operon *argCJBD* from *C. glutamicum* ATCC 21831. This strain produced 51.5 g/L ʟ-ornithine from glucose in a fed-batch culture in a 6.6-L fermenter [12]. Jiang et al. engineered a *C. glutamicum* ATCC 13032-derived strain that produced 24.1 g/L ʟ-ornithine in shake flask cultures by genetic modulation and adaptive evolution [13].

**Fig. 1:**
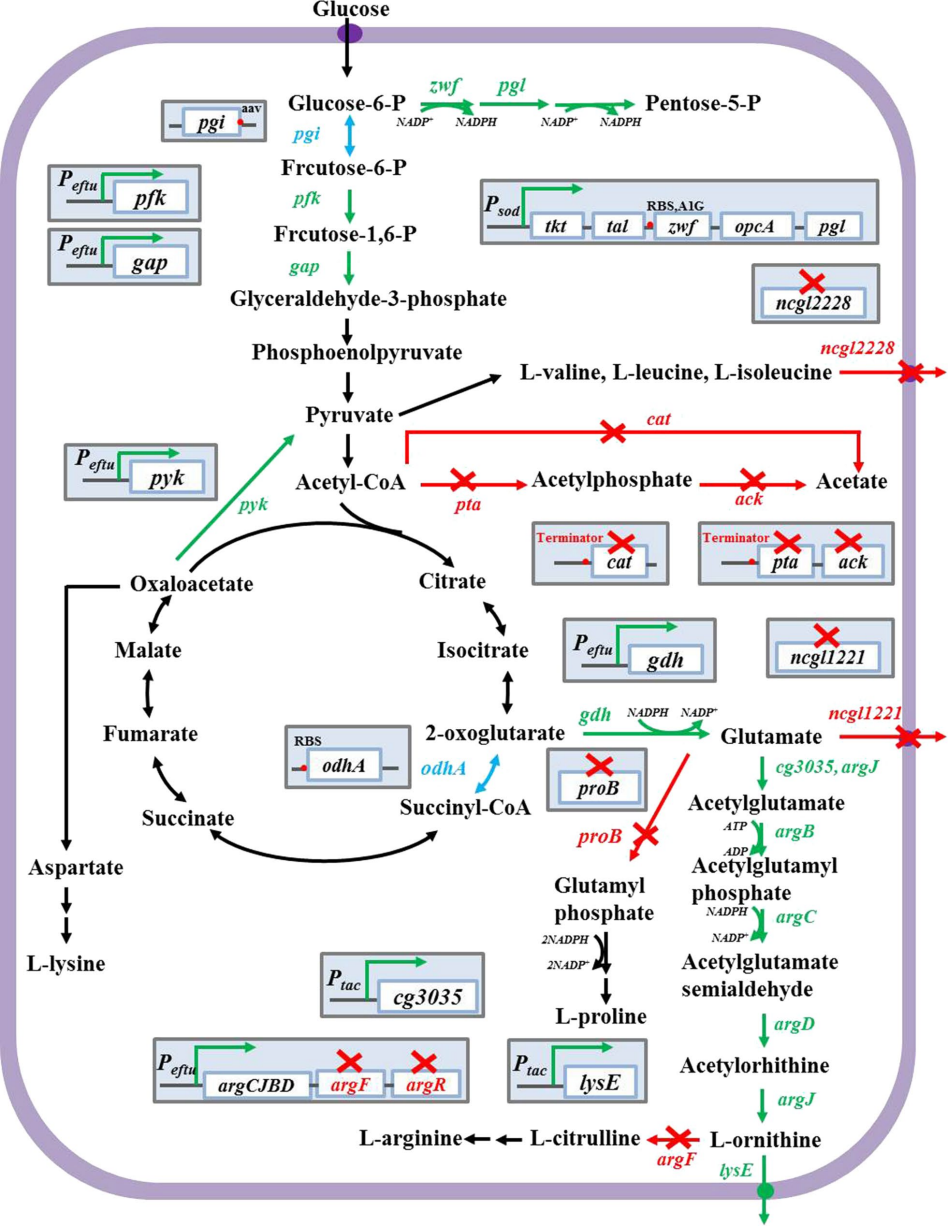
ʟ-Ornithine biosynthesis metabolic pathways in *C. glutamicum* and strategies to improve ʟ-ornithine accumulation. The red × represented this pathways were inactivated. The blue font and arrows represented that pathways were attenuated. The green font and arrows indicated that pathways were overexpressed. The genes encoding enzymes involved in catalytically relevant reactions. *pgi*, encodes glucose-6-phosphate isomerase; *zwf*, encodes glucose-6-phosphate dehydrogenase; *pgl*, encodes 6-phosphogluconolactonase; *tkt*, encodes transketolase; *tal*, encodes transaldolase; *opcA*, encodes the putative glucose-6-phosphate dehydrogenase assembly protein; *pfkA*, encodes ATP-dependent phosphofructokinase; *gap*, encodes glyceraldehyde 3-phosphate dehydrogenase; *pyk*, encodes pyruvate kinase; *pta*, encodes phosphotransacetylase; *ackA*, encodes acetate kinase; *cat*, encodes succinyl-CoA:acetate CoA-transferase; *ncgl2228*, encodes a putative branched amino acid transporter protein; *odhA*, encodes a subunit of 2-ketoglutarate dehydrogenase; *gdh*, encodes glutamate dehydrogenase; *ncgl1221*, encodes glutamate transporter; *proB*, encodes gamma-glutamate kinase; *cg3035*, encodes N-acetylglutamate synthase; *argB*, encodes N-acetylglutamate kinase; *argC*, encodes *N*-acetyl-gamma-glutamylphosphate reductase; *argD*, encodes acetylornithine aminotransferase; *argF*, encodes ornithine carbamoyltransferase; *argJ*, encodes ornithine acetyltransferase; *argR*, encodes arginine repressor; *lysE*, encodes lysing transporter

In previous study, we developed an engineered strain of *C. glutamicum* S9114 by deletion of *argF, ncgl1221, argR,* and *putP,* attenuation of *odhA*, *proB*, and *ncgl2228*, and overexpression of *lysE*, *gdh*, and *argCJBD*, which produced up to 25 g/L of ʟ-ornithine in a shake flask culture [14, 15]. In the present study, further manipulation to improve ʟ-ornithine production by the genetically engineered *C. glutamicum* strain was reported (see in Fig. 1). Overexpression of *cg3035* using plasmid-based overexpression and promoter insertion experiments for improving ʟ-ornithine production in *C. glutamicum* SO1 was explored. In addition, the effects of overexpressing the key enzymes in glycolysis, along with attenuation of acetate biosynthesis were evaluated. Next, redirection of the metabolic flux to pentose phosphate pathway was also modulated.

## Methods

### Strains and plasmids

A high ʟ-ornithine producing strain *C. glutamicum* SO1 (S9114 with deletion of *argF, ncgl1221, argR, and putP,* attenuation of *odhA*, *proB*, and *ncgl2228*, and overexpression of *lysE*, *gdh*, and *argCJBD*) derived from *C. glutamicum* S9114 was used as the original strain for further strain development. The recombinant strains and the plasmid constructed in this study are listed in Table 1. For standard molecular manipulation, *E. coli* DH5α was used as the host for gene cloning. Luria–Bertani (LB) medium was used to propagate *E. coli* and *C. glutamicum*.

**Table 1:** Strains and plasmids used in this study

Strain/plasmid	Characteristic	Source
Strain		
*E. coli* DH5ɑ	Clone host strain	Transgen
SO1	*C. glutamicum* S9114 with *deletion of argF, ncgl1221, argR, putP, attenuation of odhA, proB, ncgl2228, overexpression of lysE, gdh, argCJBD*	Lab stock
SO2	SO1 carrying expression vector pEC-XK99E	This study
SO3	SO1 carrying expression vector pEC-*cg3035*	This study
SO4	SO1 with P_*tac*_ promoter inserted in front of *cg3035*	This study
SO5	SO1 with terminator inserted in front of *pta*	This study
SO6	SO1 with terminator inserted in front of *cat*	This study
SO7	SO1 with P_*eftu*_ promoter inserted in front of *pfkA*	This study
SO8	SO1 with P_*eftu*_ promoter inserted in front of *gap*	This study
SO9	SO1 with P_*eftu*_ promoter inserted in front of *pyk*	This study
SO10	SO1 with P_*eftu*_ promoter inserted in front of *pfkA* and P_*tac*_ promoter inserted in front of *cg3035*	This study
SO11	SO1 with P_*eftu*_ promoter inserted in front of *pfkA*, P_*tac*_ promoter inserted in front of *cg3035* and a terminator inserted in front of *pta*	This study
SO12	SO1 with P_*eftu*_ promoter inserted in front of *pfkA*, P_*tac*_ promoter inserted in front of *cg3035* and a terminator inserted in front of *cat*	This study
SO13	SO1 with P_*eftu*_ promoter inserted in front of *pfkA*, P_*tac*_ promoter inserted in front of *cg3035* and terminator inserted in front of *pta and cat*	This study
SO14	SO12 with an AAV degradation label inserted in the C terminal of *pgi*	This study
SO15	SO14 with synthetic RBS and A1G replacement in front of *zwf*.	This study
SO16	SO15 with P_*tac*_ promoter inserted in front of *tkt* operon.	This study
Plasmid		
pK18*mobsacB*	Mobilizable vector, allows for selection of double crossover in *C. glutamicum*, Km^R^, *sacB*	[16]
pEC-XK99E	A shuttle expression vector, Km^R^	Lab stock
pEC-*cg3035*	A derivative of pEC-XK99E, harboring *cg3035* gene from *C. glutamicum* S9114 under its native promoter	This study
pK18-P_*tac*_-*cg3035*	A derivative of pK18*mobsacB*, harboring P_*tac*_-*cg3035* fragment	This study
pK18-T-*pta*	A derivative of pK18*mobsacB*, harboring T-*pta* fragment	This study
pK18- T-*cat*	A derivative of pK18*mobsacB*, harboring T-*cat* fragment	This study
pK18-P_*eftu*_-*pfkA*	A derivative of pK18*mobsacB*, harboring P_*eftu*_-*pfkA* fragment	This study
pK18-P_*eftu*_-*gap*	A derivative of pK18*mobsacB*, harboring P_*eftu*_-*gap* fragment	This study
pK18-P_*eftu*_-*pyk*	A derivative of pK18*mobsacB*, harboring P_*eftu*_-*pyk* fragment	This study
pK18- *pgi*-*aav*	A derivative of pK18*mobsacB*, harboring *pgi*-AAV fragment	This study
pK18-SB-*zwf*	A derivative of pK18*mobsacB*, harboring SB-*zwf* fragment	This study
pK18-P_*tac*_-*tkt*	A derivative of pK18*mobsacB*, harboring P_*tac*_-*tkt* fragment	This study

Superscript ‘‘R’’ indicates resistance to the following antibiotics: *Km* kanamycin

### DNA manipulation and strain construction

To construct the recombinant strains, the genomic DNA of strain *C. glutamicum* S9114 was isolated using a genomic DNA extraction kit (Tiangen, Beijing, China) and employed for DNA fragment amplification. The PCR product and vectors were obtained using the PCR products Purification kit and the mini-plasmid isolation kit (Tiangen, Beijing, China), respectively. The primers used in this study are listed in Additional file 1: Table S1. For introducing modifications into the chromosome of *C. glutamicum* S9114, the suicide vector pK18*mobsacB* containing the sucrose lethal gene *sacB* from *Bacillus subtilis* was employed by double crossover recombination, as described previously [16, 17].

For *cg3035* overexpression in the high ʟ-ornithine producing strain SO1, a widely used strong P_*sod*_ promoter and the open reading frame of *cg3035* was amplified and spliced using PCR. The overlapped fragment was ligated into the expression vector pEC-XK99E by Gibson assembly to construct a constitute expression cassette pEC-*cg3035*. After isolation from *E. coli*, this recombinant plasmid and empty vector pEC-XK99E were transformed into SO1 by electroporation. The positive transformants were pointed out and confirmed by colony PCR.

For P_*tac*_ promoter insertion in front of *cg3035*, the upstream region and the coding region of *cg3035* was amplified. A strong P_*tac*_ promoter was introduced between the upstream region and coding region by primers. The overlapping fragment containing the upstream region, P_*tac*_ promoter, and the coding region was cloned into the *Hin*d III/*Xba* I sites in the suicide vector pK18*mobsacB* by Gibson assembly. The engineered plasmid was then transferred into strain SO1 by electroporation. After double crossover recombination, the positive recombinant strain was detected by colony PCR. Similarly, the insertion of the P_*eftu*_ promoter in front of *pfkA*, *gap* and *pyk* or insertion of P_*tac*_ in front of *tkt* was also performed using these procedures.

To attenuate the expression of genes, strategy described in our previous study were carried out [18]. To attenuate the expression of *pta* and *cat*, a transcription terminator was introduced into the right upstream of the genes *pta* and *cat* which lines between the upstream fragment and downstream fragment by PCR. The recombinant fragment was then cloned into the *Hind* III/*Xba* I sites in the suicide vector pK18*mobsacB* by Gibson assembly. After extraction from *E. coli*, this engineered vector was transformed into SO1 by electroporation. After double crossover recombination, the mutant strains with a terminator inserted in the upstream region of *pta* and *cat* were determined by colony PCR. When constructing the mutant strains, an AAV taq was inserted in the C terminal of *pgi* with a synthetic RBS and G1A replacement in *zwf*. The RBS sequence with the predicted translation start strength of 50000 au was designed using the RBS Calculator (https://www.denovodna.com/software/doLogin) and is listed in Additional file 1: Table S2.

### Fermentation in shake flasks

Fermentation with recombinant strains was performed in batch culture in shake flasks as described in our previous work [14, 15]. A single clone of the mutants was activated on LB agar plate for two cycles of 12 h. Subsequently, a ring of bacteria was inoculated into 10 mL of seed medium in a 100-mL normal shake flask. The seed medium consisted of (per liter) 25 g glucose, 10 g yeast extract, 10 g corn steep liquor, 15 g (NH_4_)_2_SO_4_, 2.5 g MgSO_4_·7H_2_O, 1 g KH_2_PO_4_, 0.5 g K_2_HPO_4_, 0.5 g Na_2_HPO_4_, and 10 g CaCO_3_. After 11 h of cultivation at 32 °C and 220 rpm, the appropriate amount of culture was transferred to 24 mL of fermentation medium in a 250-mL baffle shake flask. Initial OD_600_ of the fermentation culture was adjusted to one. The fermentation medium consisted of (per liter) 100.0 g glucose, 20.0 g corn steep liquor, 50.0 g (NH_4_)_2_SO_4_, 2.5 g MgSO_4_·7H_2_O, 1.0 g KH_2_PO_4_, 0.5 g K_2_HPO_4_, 0.5 g Na_2_HPO_4_, 0.02 g FeSO_4_·7H_2_O, 0.02 g MnSO_4_·4H_2_O, and 10 g CaCO_3_. The initial pH was adjusted to 7.0. All cultures were grown at 32 °C and 250 rpm, and 200-µL samples were collected every 12 h to measure l-ornithine concentration, cell density, and residual glucose concentration. If necessary, 50 mg/L kanamycin was used to cultivate *E. coli* and 12.5 mg/L kanamycin was used to cultivate *C. glutamicum*.

### Measurement of NAGS enzyme activity

For NAGS enzyme activity analysis, 20 mL of fermentation samples were collected at 10 h by centrifugation (at 5000 rpm, 4 °C, and 10 min) and washed twice with 100 mM Tris–HCl (pH 7.5) which was supplemented with 20 μM PMSF. Following the pure cells were incubated in 5 mL of 100 mM Tris–HCl (pH 7.5) containing 30% (v/v) glycerol and 10 mg/mL lysozyme at 37 °C for 3 h and then disrupted by sonication. After removing cell debris by centrifugation, the supernatant was collected as crude enzyme, and the protein concentration was determined by bicinchoninic acid (BCA) assay using bovine serum albumin as the standard. NAGS specific activity assay was performed as described previously [19].

### RT-PCR

For RNA analysis, 500 μL of fermentation samples were collected at 12 h. RNA extraction and RT-PCR assays were performed as described in our early report [20].

### Measurement of glucose consumption, optical density, and metabolite analysis

After dissolving CaCO_3_ in 0.125 mol/L HCl, cell growth was monitored by measuring the OD_600_ using a microplate reader (BioTek Instruments, Winooski, VT, USA). ʟ-Ornithine concentrations were determined by colorimetry using ninhydrin, as described previously [21, 22]. l-Ornithine standard curve of colorimetric assay was listed in Additional file 1: Figure S1. The fermentation supernatant was passed through a 0.22-µm filter and analyzed for glucose levels, using a SBA-40C biosensor (developed by Biology Institute of Shandong Academy of Sciences). Acetic acid were analyzed as described previously [23]. All experiments were conducted in triplicate; the data were averaged and presented as mean ± standard deviation (SD).

## Results

### Overexpression of cg3035 exerts a positive effect on ʟ-ornithine production

**Fig. 2:**
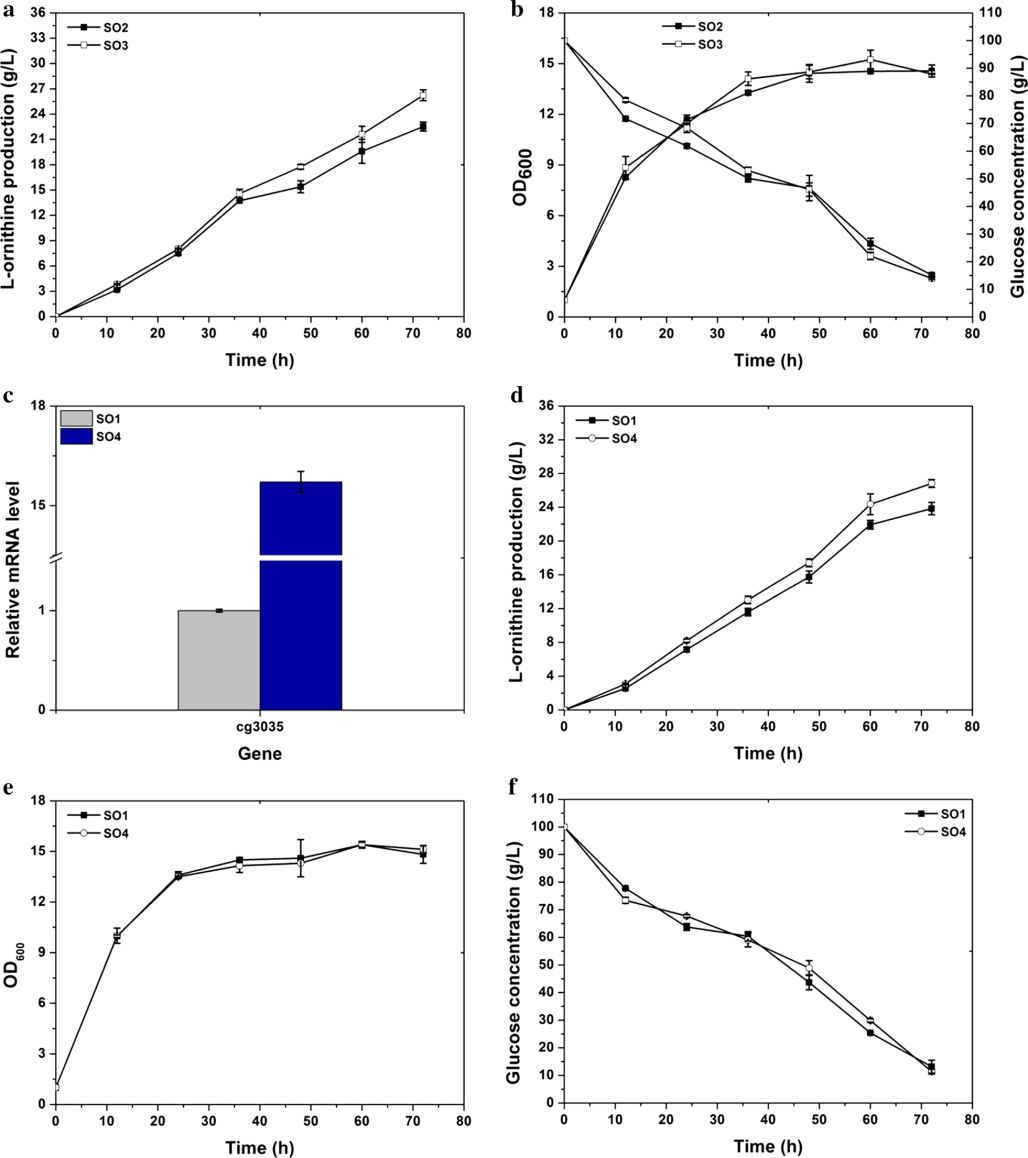
Improvement of ʟ-ornithine production by *cg3035* overexpression. **a** ʟ-Ornithine production curves for strains SO2 (SO1 carrying pEC-XK99E) and SO3 (SO1 carrying pEC-*cg3035*). **b** Cell growth and glucose consumption of strains SO2 and SO3. **c** Relative transcription level of *cg3035* in engineered strains SO1 and SO4 (SO1 with P_*tac*_ promoter inserted upstream of *cg3035*). **d** ʟ-Ornithine production curves for the engineered strains SO1 and SO4. **e** Cell growth. **f** Glucose consumption. Samples were collected per 12 h for determining fermentation parameters. Results of standard deviations present in three individual experiments

Glutamate acetylation is the first step in the conversion of glutamate to ʟ-ornithine and prevents glutamate from cyclisation and further conversion to ʟ-proline. Petri et al. reported that glutamate acetylation is catalyzed by *cg3035*, which updated the previous knowledge that *argJ* encodes the bifunctional enzyme for glutamate acetylation and *N*-acetylornithine deacetylation [19]. In a previous study, we constructed a high ʟ-ornithine producing strain *C. glutamicum* strain SO1 (S9114 with deletion of *argF*, *ncgl1221*, *argR*, and *putP*, attenuation of *odhA*, *proB*, and *ncgl2228*, and overexpression of *lysE*, *gdh*, and *argCJBD*) and found that *argCJBD* expression was not the rate-limiting step for ʟ-ornithine accumulation. Therefore, glutamate acetylation prevented further improvement of ʟ-ornithine accumulation. To overcome this barrier, *cg3035* was overexpressed using an expression vector pEC-XK99E in the engineered strain SO1, thus generating strain SO3. Compared with strain SO1, NAGS enzyme activity in strain SO3 was improved by 5.63-fold, which suggested that *cg3035* was successful overexpressed. The empty plasmid pEC-XK99E was also transformed into strain SO1 to produce strain SO2 as a control. To fully evaluate the performance of the engineered strains SO2 and SO3, shake flask fermentation was performed. After 72 h of cultivation, the mutant strain SO3 produced 26.2 g/L of ʟ-ornithine, which is 16.5% higher than that (22.5 g/L) obtained with strain SO2 (Fig. 2a). The cell growth of strain SO3 as well as its glucose consumption was comparable to that of the control strain SO2; indicating *cg3035* overexpression is nontoxic to *C. glutamicum* (Fig. 2b). These results confirmed our speculation that glutamate acetylation plays an important role in ʟ-ornithine biosynthesis. Inspired by the results of plasmid-based *cg3035* overexpression, a strong mutant P_*tac*_ promoter developed by Jakob [24] was integrated into the chromosome of the parent strain SO1 to address the challenges associated with genetic instability using a vector, to generate strain SO4. This strategy was successfully applied fsor overexpressing *lysE* during our previous work. The relative mRNA level of *cg3035* and NAGS enzyme activity in strain SO4 were 14.7-fold and 3.62-fold higher than that in the control strain, respectively, which illustrated that the expression of *cg3035* was effectively strengthened through the insertion of P_*tac*_ promoter (Fig. 2c and Table 2). During 72 h of fermentation, the ʟ-ornithine production titer of strain SO4 was measured to be 26.8 g/L, which was increased by 12.6% compared to that of the parent strain SO1 (23.8 g/L) (Fig. 2d). Similar to the result obtained by using a vector for *cg3035* overexpression, the engineered strain SO4 exhibited coincident growth and glucose consumption as the control strain SO1 (Fig. 2e, f). In consequence, these results provide detailed knowledge of *cg3035* expression to improve ʟ-ornithine accumulation.

**Table 2:** Acetic acid concentration in 72 h fermentation broth

Strains	Relative NAGS specific activity	Acetic acid concentration (g/L)
SO1	1 ± 0.13	3.85 ± 0.14
SO3	6.63 ± 0.07	–
SO4	4.62 ± 0.21	–
SO5	–	1.05 ± 0.05
SO6	–	1.34 ± 0.06

### Improvement of ʟ-ornithine production by attenuating the acetate synthesis pathway

**Fig. 3:**
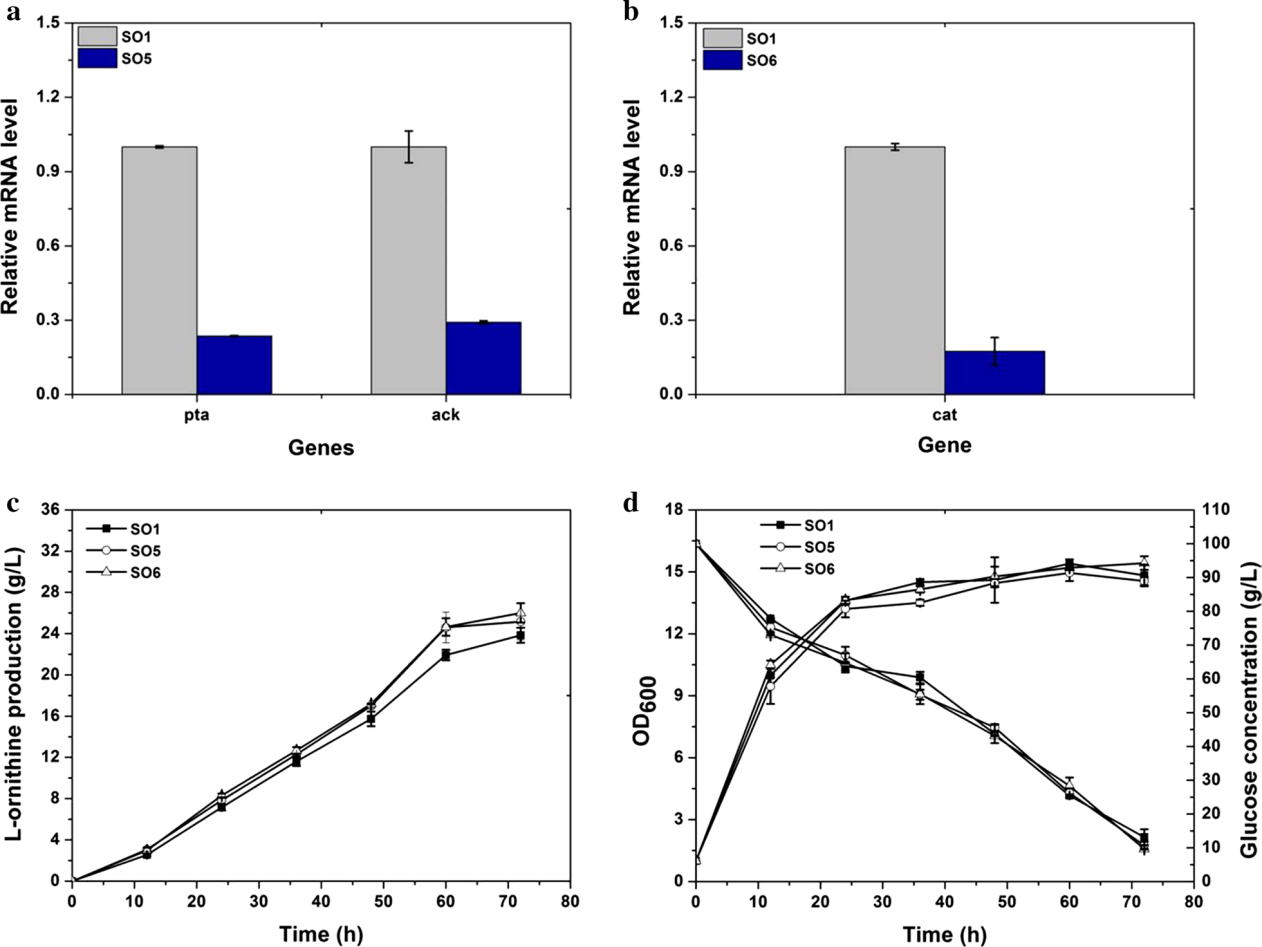
Blocking acetate biosynthesis by inserting a terminator into the upstream region of *pta* and *cat*, and its effect on ʟ-ornithine production. **a** Relative *pta* and *ack* mRNA levels in SO1 and SO5 (SO1 carrying modulation in *pta*). Samples at the 12 h time point were subjected to transcript analysis during fermentation cultivation. **b** Relative *cat* mRNA levels in SO1 and SO6 (SO1 carrying modulation in *cat*). **c** ʟ-Ornithine production by the engineered strains SO1, SO5, and SO6. **d** Cell growth and glucose consumption. Samples were collected per 12 h for determining the fermentation parameters. Results of standard deviations present in three individual experiments

Glutamate acetylation, a confirmed rate-limiting step for further improvement of ʟ-ornithine production in engineered *C. glutamicum* SO1, is the first reaction in the ʟ-ornithine pathway that uses acetyl-CoA as second substrate next to glutamate. We speculated that insufficient acetyl-CoA supplementation hindered the biosynthesis of ʟ-ornithine in these strains. To spare acetyl-CoA for ʟ-ornithine biosynthesis, we considered acetic acid biosynthesis that consumes acetyl-CoA and consists of two synthetic branches catalyzed by the products of *pta* and *ack*, or *cat*. Therefore, we attenuated these two acetate synthetic branches by insertion of a terminator, which was used to attenuate the expression of *proB* and *ncgl2228* in previous work, in the upstream region of these genes, thus generating strains SO5 and SO6. Considering that *pta* and *ack* are listed as an operon, *ack* expression will also be attenuated by inserting a terminator in the upstream region of *pta*. The performance of these two strains was evaluated by shake flask fermentation. As shown in Fig. 3a, b, the expression of *pta* and *ack* in the mutant strain SO5 and the relative mRNA level of *cat* in the engineered strain SO6 was significantly reduced compared with that in the parental strain SO1. In addition, the yield of acetic acid produced by strain SO5 and SO6 were also reduced approximately 2.5-fold than strain SO1 (Table 2). This indicated that insertion of a terminator upstream of *pta* and *cat* was an efficient strategy to downregulate their expression. Strain SO5 and SO6 produced 25.1 and 26 g/L of ʟ-ornithine, which was 5.5 and 9.1% higher than that of the control strain SO1 (23.8 g/L) (Fig. 3c). Results obtained per 12 h represent a significant improvement of ʟ-ornithine synthesis compared with that of the control strain. Despite altered acetate biosynthesis, the engineered strains SO5 and SO6 grow robustly in the fermentation medium, maintaining a stable rate of glucose consumption (Fig. 3d). These results indicate that attenuating the expression of genes involved in acetate biosynthesis is a viable strategy for improving ʟ-ornithine accumulation.

### Effect of overexpressing enzymes of the glycolytic pathway on ʟ-ornithine production

**Fig. 4:**
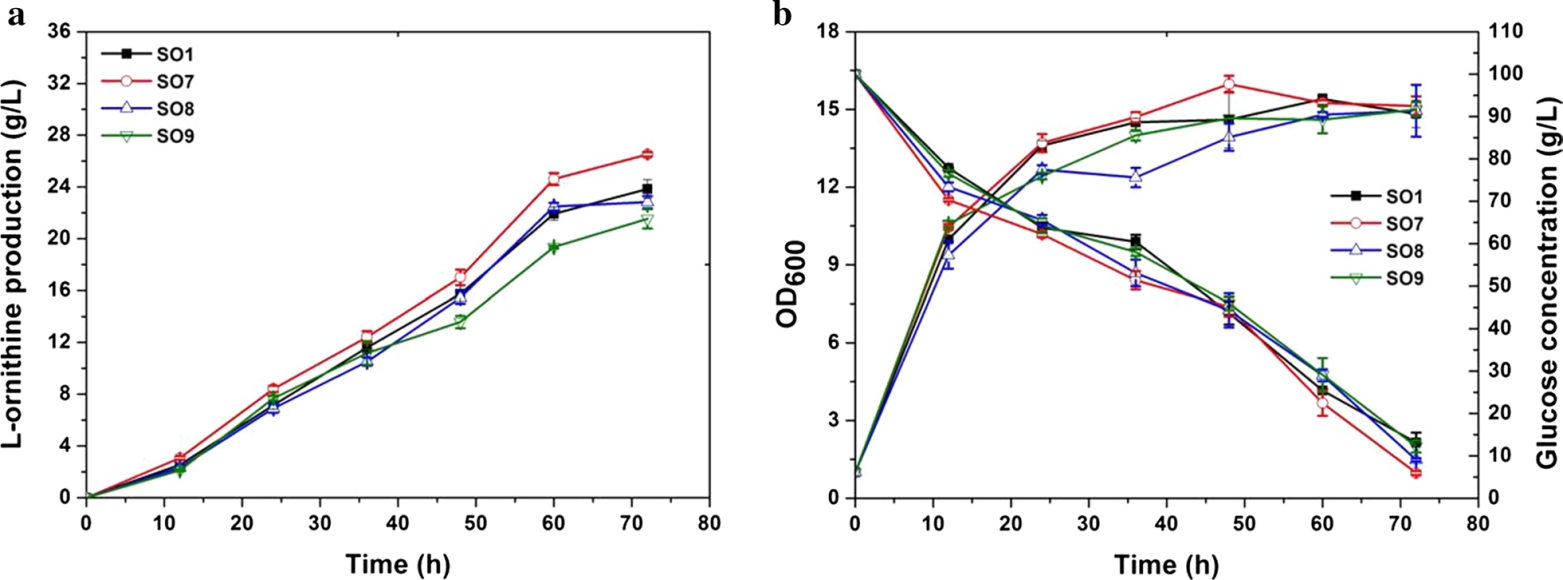
Assessment of ʟ-ornithine productivity and other characterizations of strains SO1, SO7 (SO1 carrying P_*eftu*_ promoter inserted upstream of *pfkA*), SO8 (SO1 with P_*eftu*_ promoter inserted upstream of *gap*), and SO9 (SO1 with P_*eftu*_ promoter inserted upstream of *pyk*). **a** ʟ-Ornithine production curves for strains SO1, SO7, SO8, and SO9. **b** Cell growth and glucose consumption. Samples were collected per 12 h for fermentation parameter determination. Data represent average values and standard deviations from three individual experiments

As previously demonstrated, plasmid-based overexpression of *gap* encoding NAD-dependent glyceraldehyde-3-phosphate dehydrogenase, *pfkA* encoding 6-phosphofructokinase, and *pyk* encoding pyruvate kinase was able to improve ʟ-ornithine production in the engineered *C. glutamicum* ATCC 13032 strain [22]. Therefore, to examine if enhancing the expression of these genes in strain SO1 could further improve ʟ-ornithine production, we inserted a strong P_*eftu*_ promoter into the upstream region of *pfkA, gap,* and *pyk* in strain SO1, to construct strains SO7, SO8, and SO9, respectively. After cultivation in the fermentation media for about 72 h, strains SO7, SO8, and SO9 produced 26.5, 22.8, and 21.5 g/L of ʟ-ornithine (Fig. 4a). The yield of ʟ-ornithine produced by strain SO7 with *pfkA* overexpression was 11.2% higher than that of the parental strain SO1. Strain SO8 with *gap* overexpression produced the same amount of ʟ-ornithine as that of the parent strain SO1. The yield of ʟ-ornithine produced by strain SO9 with P_*eftu*_ inserted in front of *pyk* was decreased by 9.7% compared to strain SO1. The growth of strain SO8 and SO9 was slower than that of the engineered strains SO1 and SO7. Glucose consumption by strain SO7 was slightly faster than that by strains SO1, SO8, and SO9. Taken together, those results suggest that modulation of *pfkA* promotes ʟ-ornithine accumulation in engineered *C. glutamicum* S9114.

### Combination of P_tac_-cg3035, P_eftu_-pfkA, T-pta, and T-cat targets and its influence on ʟ-ornithine production

**Fig. 5:**
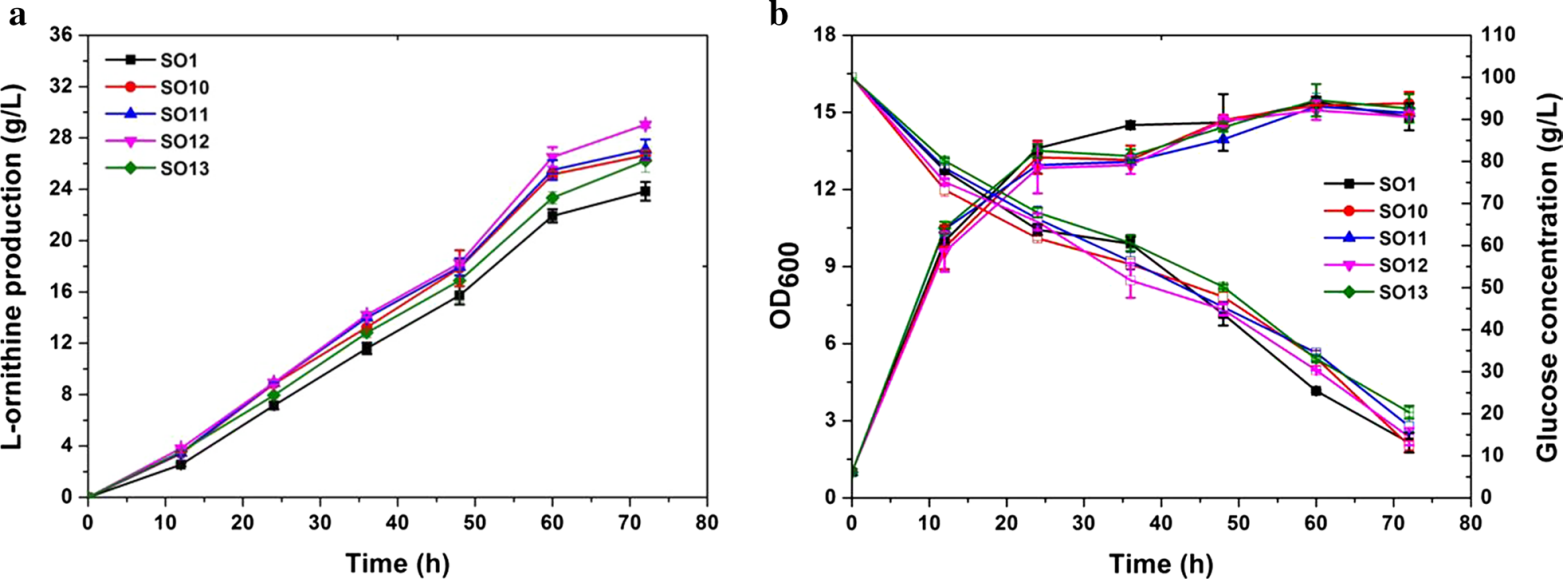
ʟ-Ornithine productivity, cell growth, and glucose consumption during cultivation of SO1, SO10 (SO1 carrying P_*eftu*_ promoter inserted upstream of *pfkA* and P_*tac*_ promoter inserted upstream of *cg3035)*, SO11 (SO1 carrying P_*eftu*_ promoter inserted upstream of *pfkA*, P_*tac*_ promoter inserted upstream of *cg3035* and terminator inserted in the upstream region of *cat*), SO12 (SO1 carrying the P_*eftu*_ promoter inserted upstream of *pfkA*, P_*tac*_ promoter inserted upstream of *cg3035*, and a terminator inserted upstream region of *pta*), and SO13 (SO1 carrying the P_*eftu*_ promoter inserted upstream of *pfkA*, P_*tac*_ promoter inserted upstream of *cg3035*, and a terminator inserted in the upstream region of *pta* and *cat*). **a** ʟ-Ornithine production curves for strains SO1 (black square), SO10 (red circle), SO11 (blue upper triangle), SO12 (pink lower triangle), and SO13 (green quadrangle). **b** Cell growth and glucose consumption. Samples were collected per 12 h for fermentation parameter determination. Data represent the average values and standard deviations from three individual experiments

From the above results, several efforts including independently inserting a P_*tac*_ in front of *cg3035*, inserting a P_*eftu*_ in front of *pfkA*, and inserting a terminator in the upstream region of *pta* or *cat* were observed to enhance ʟ-ornithine accumulation. Since our major goal is to construct a high ʟ-ornithine producing strain, we combined these targets and constructed strain SO10 carrying *P*_*tac*_-*cg3035* and *P*_*eftu*_-*pfkA*, SO11 with *P*_*tac*_-*cg3035*, *P*_*eftu*_-*pfkA*, and *T*-*cat,* SO12 with *P*_*tac*_-*cg3035*, *P*_*eftu*_-*pfkA*, and *T*-*pta*, and SO13 with *P*_*tac*_-*cg3035*, *P*_*eftu*_-*pfkA*, *T*-*pta*, and *T*-*cat*. Next, the effects of these modifications on ʟ-ornithine accumulation were tested in the context of shake flask fermentation. As shown in Fig. 5a, mutant strains incorporating the modulation of these targets produced more ʟ-ornithine compared to the parent strain SO1. The highest ʟ-ornithine production titer in 72 h cultivation was produced by strain SO12 and reached 29 g/L, which was 21.8% higher than that obtained with strain SO1 (23.8 g/L). Unexpectedly, strain SO13, which possessed all of these modifications, exhibited a lower ʟ-ornithine production performance and relatively slow glucose utilization efficiency compared to strain SO12 (Fig. 5b). In conclusion, these results illustrate that these targets exhibit a positive synergistic effect on improving ʟ-ornithine production.

### Enhancement of ʟ-ornithine production by redirecting the metabolic flux into pentose phosphate pathway

**Fig. 6:**
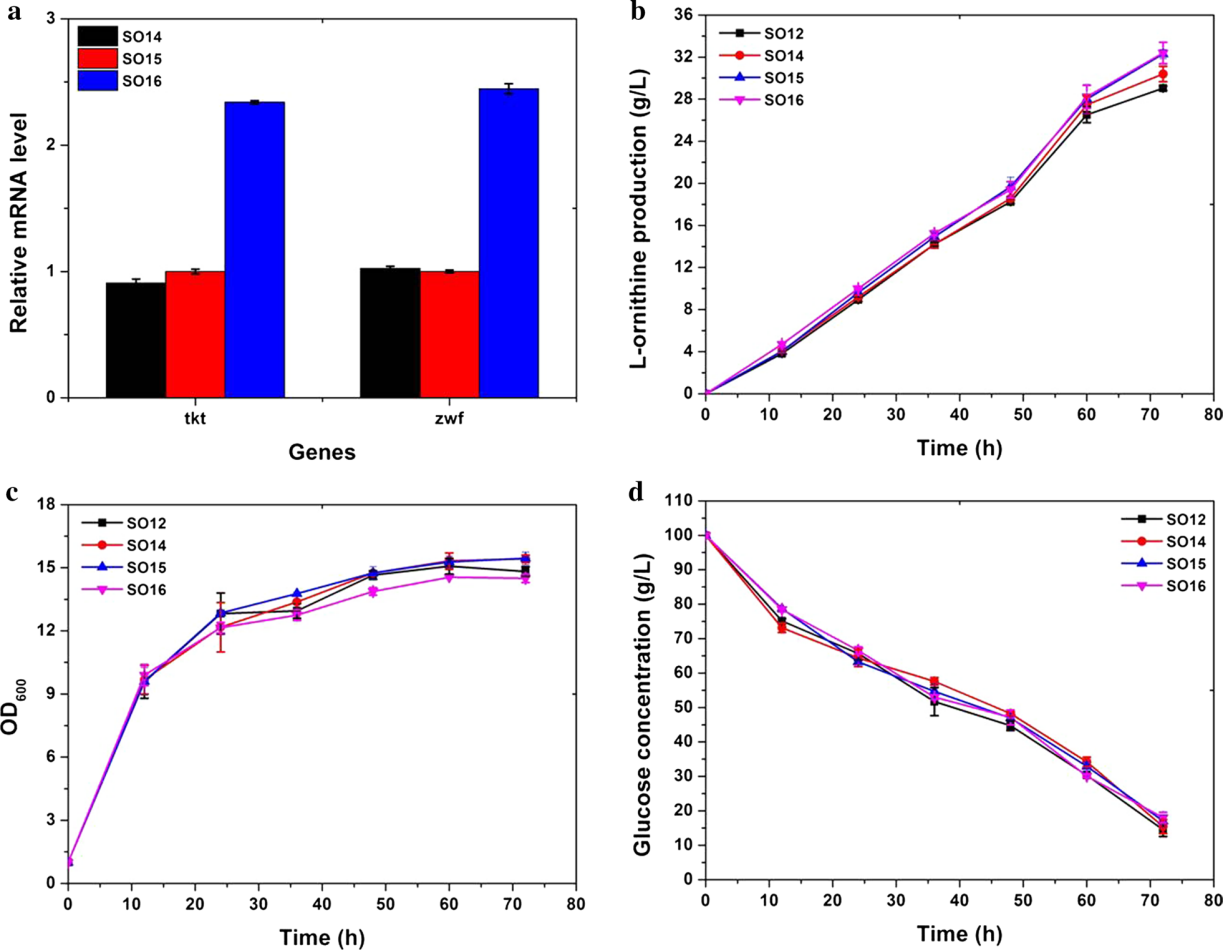
Pentose phosphate pathway modulation and its effect on ʟ-Ornithine production. **a** Relative *tkt* and *zwf* mRNA levels in strains SO15 (SO12 carrying AAV taq inserted into the C-terminal of Pgi and RBS together with start codon T1A replacement in front of *zwf*) and SO16 (SO15 carrying P_*tac*_ promoter inserted in front of *tkt* operon). Samples at the 12 h time point were subjected to transcript analysis during fermentation cultivation. **b** ʟ-Ornithine production curves for strains SO12 (black square), SO14 (SO12 carrying AAV taq inserted into the C-terminal of Pgi) (red cycle), SO15 (blue upper triangle), and SO16 (pink lower triangle). **c** Growth profiles of these strains. **d** Glucose consumption curves. Samples were collected per 12 h for fermentation parameter determination. Data represent the average values and standard deviations from three individual experiments

ʟ-Ornithine biosynthesis in *C. glutamicum* requires 2 mol of NADPH. Improving NADPH supplementation is an important factor for ʟ-ornithine accumulation. Several strategies such as redirecting carbon flux to the pentose phosphate pathway, overexpression of *Clostridium acetobutylicum gapC*, deletion of putative oxidoreductases, and so on for enhancing NADPH supplementation were confirmed to increase the production titer of ʟ-ornithine. Nevertheless, as NADPH supply is frequently described as a limiting factor for ʟ-ornithine biosynthesis and the engineered strain SO13 does not carry any genetic modification to enhance NADPH supply, the question of whether NADPH supply is limiting or not remains open. Therefore, we redirected the carbon flux to the pentose phosphate pathway through attenuation of *pgi*, overexpression of the *tkt* operon, and changing the RBS and translation start codon of *zwf* in the engineered strain SO12, to generate strains SO14 (SO12 with an AAV taq inserted in the C-steam of *pgi*), SO15 (SO14 with a strong P_*tac*_ promoter inserted in front of the *tkt* operon), and SO16 (SO15 with G1A and RBS replacement in *zwf*). The relative *tkt* and *zwf* mRNA levels in strain SO16 were approximately 1.5-fold higher than those in strain SO15, indicating that insertion of the P_*tac*_ promoter is able to improve the transcription level of the *tkt* operon (Fig. 6a). Fermentation in a shake flask revealed that strains SO14, SO15, and SO16 produced 30.38, 32.30, and 32.38 g/L ʟ-ornithine with productivities of 0.359, 0.389, and 0.395 g/g glucose, respectively, which is 4.5, 11.4, and 11.4% higher than that of strain SO12 (29 g/L). Compared with strain SO12, SO14, and SO15, strain SO16 exhibited a slightly lower growth (Fig. 6c). Strain SO14 demonstrated relatively lower glucose consumption compared to other strains (Fig. 6d). These results illustrate that improving the availability of NADPH is an inevitable modulation for ʟ-ornithine accumulation in *C. glutamicum*.

## Discussion

In this study, we demonstrate system-wide engineering and optimization of cellular metabolism in *C. glutamicum* SO1 to further improve strain performance with respect to ʟ-ornithine biosynthesis. Depending on the current knowledge about ʟ-ornithine biosynthesis pathway, *cg3035* was overexpressed using a plasmid that significantly improved ʟ-ornithine production. This confirmed our speculation that glutamate acetylation is a rate-limiting step for ʟ-ornithine accumulation after removing the feedback inhibition of ArgR on the *argCJBDF* operon. In the incipient fermentation period, the improvement of ʟ-ornithine production was not obvious probably due to slow glucose utilization rate. In addition, we induced genome-based *cg3035* overexpression to ensure strain stability, which also promote ʟ-ornithine accumulation. The improved percentage in ornithine production by genome-based *cg3035* overexpression was lower than plasmid based *cg3035* overexpression probably due to the not superior NAGS enzyme activity. We believe that this intriguing target could be extended to ʟ-arginine and ʟ-citrulline production.

Notably, *N*-acetylglutamate biosynthesis also requires acetyl-CoA as a precursor in addition to N-acetylglutamate synthase. Therefore, we attenuated the acetate biosynthesis pathway to reduce acetyl-CoA consumption and improved ʟ-ornithine production titer, thus indicated that acetyl-CoA supplementation is an important factor for ʟ-ornithine production, which further confirmed previous reports that deletion of *dtsR1* encoding a subunit of acetyl-CoA carboxylase is beneficial for ʟ-glutamate [25] and ʟ-arginine accumulation [26].This result is also consistent with previous work reported that block the acetate biosynthesis pathway promote the production of several compounds [27–30]. Attenuating acetate biosynthesis also conserves carbon flux into the glycolytic pathway, which may also account for the high ʟ-ornithine production performance. This encouraged us to explore the glycolytic pathways. Overexpression of *pfkA* by the insertion of a strong P_*eftu*_ promoter tremendously promoted ʟ-ornithine production, whereas insertion of a strong P_*eftu*_ promoter in front of *gap* or *pyk* did not contribute to ʟ-ornithine accumulation, which is in contrast with previous results [22]. The differences in genetic background between *C. glutamicum* ATCC 13032 and *C. glutamicum* S9114 may account for these diverse outcomes. There is a one percent gap in the genomes of those two strains. In addition, *C. glutamicum* S9114, a mutant strain derived from *Brevibacterium tianjinese* T6–13, processed a faster cell growth than *C. glutamicum* ATCC 13032. Modulations in *pyk* and *gap* slightly affected cell growth and glucose consumption in *C. glutamicum* S9114 probably acting as the main reason for the reduced ʟ-ornithine yield. Based on the aforementioned results, *P*_*tac*_-*cg3035*, *P*_*eftu*_-*pfkA*, *T*-*pta*, and *T*-*cat* were confirmed to independently increase ʟ-ornithine accumulation. To further improve the yield of ʟ-ornithine, these targets were assembled. The highest performance was obtained in strain SO12 with *P*_*tac*_-*cg3035*, *P*_*eftu*_-*pfkA*, and *T*-*pta*, but not in strain SO13 with *P*_*tac*_-*cg3035*, *P*_*eftu*_-*pfkA*, *T*-*pta*, and *T*-*cat*, which illustrated that attenuating *pta* and *cat* simultaneously can affect ʟ-ornithine production by interfering with normal physiological metabolism in engineered strains.

Several previous works have demonstrated that NADPH availability is strongly correlated with ʟ-ornithine production. In this work, ʟ-ornithine production was improved by suppositional redirecting the carbon flux to the pentose phosphate pathway according to previous study [12], which further demonstrated the importance of NADPH supplementation in developing ʟ-ornithine producing strains. This is consistent with previous work by Kim et al., where the pentose phosphate pathway was reinforced by replacing the native promoter of the tkt operon with a strong sod promoter and changing the start codons of *zwf* and *pgi* [12].

In summary, compared with previous studies shown in Table 3, this study provide more systematic and comprehensive steps in the construction of an engineered high ʟ-ornithine producing *C. glutamicum* S9114 strain. Currently, the highest ʟ-ornithine production titer (51.5 g/L) was reported by Kim et al. [12], which employ a bioreactor for fed-batch fermentation (Table 3). However, ʟ-ornithine production yield per gram glucose via fed-batch fermentation (0.24 g/g) was lower than that of shake flask batch fermentation (0.524 g/g) developed by Jensen et al. [10]. In the following work, we will try more strategies such as releasing feedback inhibition of *argB*, which is probably the main reason why ʟ-ornithine yield in this study was lower than 0.524 g/g glucose, and continue to modulate the metabolic pathway to further improve the production titer of ʟ-ornithine. In addition, inspired by previous work [12], employ a fermentation bioreactor and change the fermentation mode to fed-batch cultivation was supposed to promote ʟ-ornithine accumulation.

**Table 3:** Comparison of other *C. glutamicum* strains engineered for ʟ-ornithine production

Strains (*C. glutamicum*)	ʟ-Ornithine titer (g/L)	ʟ-Ornithine yield (g/g glucose)	Cultivation	Modulations	References
SO16	32.3	0.395	Shake flask; batch	Deletion of *argF*, *ncgl1221*, *argR*, and *putP*; attenuation of *odhA*, *proB*, *pta*, *cat* and *ncgl2228*; overexpression of *lysE*, *gdh*, *cg3035*, *pfkA*, *pyk*, *tkt*, and *argCJBD*	This study
YW06 (pSY223)	51.5	0.240	Bioreactor; fed-batch	Deletion of *argF*, *argR*, and *proB*; Reinforcement of the PPP pathway flux; The use of a feedback-resistant enzyme	[12]
ORN6	20.96^a^	0.524	Shake flask; batch	Deletion of *argF*, *argR*, and *argG*; overexpression of *argB*^*M*^; attenuation of *pgi*.	[10]
SJC8039 ∆*ncgl0281*∆*ncgl2582*∆*ncgl2053*	14^a^	ND	Shake flask; batch	Deletion of *argF*, *argR*, and *proB*; Blocking gluconate biosynthesis	[11]
∆APE6937R42	24.1	0.298	Bioreactor; batch	Deletion of *argF*, *argR*, and *proB*; Adaptive evolution in presence of ʟ-ornithine	[13]
1006∆*argR*-*argJ*	31.6	0.396	Shake flask; batch	Deletion of *argR*; overexpression of *argJ*.	[31]

^a^These values were not described in the main text of the original reference and thus estimated from the figure or graph

## Conclusion

Redesigning and engineering strains for use in the industrial production of ʟ-ornithine has significant potential application for reducing the economic cost pressures of using ʟ-arginine for enzymatic reaction and extending the fermentation organism. *C. glutamicum* has been extensively studied for its ability to produce ʟ-ornithine, though its yield and productivity are still low compared to other strategies. Very recently, we reported a *C. glutamicum* S9114 derived recombinant strain with high ʟ-ornithine production titer. Here, we have further enhanced the ʟ-ornithine yield through genome-based *cg3035* overexpression and systematic manipulation of central metabolic pathways including glycolysis, acetate metabolism, and pentose phosphate pathway, consequently suggesting that improved ʟ-ornithine production can be obtained by these modulations. *C. glutamicum* is an excellent producer for producing ʟ-glutamate and ʟ-glutamate-derived products including ʟ-ornithine, ʟ-citrulline, and ʟ-arginine. We supposed that the metabolic engineering strategies reported in this work can be applied to constructing strains producing such products.

## Additional file

**Additional file 1: Table S1.** Primers and their sequences in this study. **Table S2.** Promoter and terminator sequence used in this study. **Figure S1.** l-Ornithine standard curve of colorimetric assay using ninhydrin.
