# Supplementary material for: Optimization of ʟ-ornithine production in recombinant Corynebacterium glutamicum S9114 by cg3035 overexpression and manipulating the central metabolic pathway
Source: Microb Cell Fact. 2018 Jun 13;17:91. doi: 10.1186/s12934-018-0940-9 (PMC6001011; doi:10.1186/s12934-018-0940-9)
Supplement: Supplementary file 1 — Additional file 1: Table S1. Primers and their sequences in this study. Table S2. Promoter and terminator sequence used in this study. Figure S1. l-Ornithine standard curve of colorimetric assay using ninhydrin. [file 12934_2018_940_MOESM1_ESM.docx]

Additional file 1: Table S1. Primers and their sequences in this study

| Primers | Sequence (5’-3’) |
| --- | --- |
| *Sod*-F (cg3035): | CGC**GAGCTC**TGCCAATTATTCCGGGCTTG |
| *Sod*-R (cg3035): | CAAACCAGGCTCATTTCCGCACCGAGCATATACATCTT |
| *cg3035*-F: | CTCGGTGCGGAAATGAGCCTGGTTTGACCTGGAT |
| *cg3035*-R: | CCC**AAGCTT**CGAGCAGAGTTGACGTTCCAA |
| *cg3035*-up-F: | aacgacggccagtgccaagctTGCATGTCCGAAGTCATGTGG |
| *cg3035*-up-R (Ptac): | AACCATTATAACACAGATTCAAATTAATGTCAACCCCTAGGTGTCATGCCCGAAAGG |
| *cg3035*-down-F(Ptac): | AATTTGAATCTGTGTTATAATGGTTAAGGAGATATACATGACACCTAGTCTTCCCCGTT |
| *cg3035*-down-R: | cggtacccggggatcctctagACAACCTGGAGATATGCCTCC |
| *pta*-up-F: | aacgacggccagtgccaagcttGAGAACACGATCGCGTCATAA |
| *pta*-up-R: | AAAAGGGCGATCATCTGACCGCCCTGAGCCGAACTCCAAGGTCTG |
| *pta*-down-F: | GGGCGGTCAGATGATCGCCCTTTTTTTTTTACCTGCCCGACGAAGAACTA |
| *pta*-down-R: | cggtacccggggatcctctagGTTCGGGTTAACAGCACAGTC |
| *cat*-up-F： | aacgacggccagtgccaagcttTCCGCCAAGTTTTCATCAACG |
| *cat*-up-R: | AAAAGGGCGATCATCTGACCGCCCTCCCGCACTGCTATACCCAAG |
| *cat*-down-F: | GGGCGGTCAGATGATCGCCCTTTTTTTTTTGTCTGATCGCATTGCTTCAG |
| *cat*-down-R: | cggtacccggggatcctctagGGTGTAGGCCTGGATGTTCTC |
| *pfkA*-up-F: | aacgacggccagtgccaagctAAGCGGGAGTTCAATGAGCAA |
| *pfkA*-up-R: | CACAGATTCAAATTAATGTCAACCCAGAAAATCACAGGGGTTGACC |
| P*_eftu_*-F(pfkA): | GGGTTGACATTAATTTGAATCTGTGCACAGGGTAGCTGGTAGTTTG |
| P*_eftu_*-R(pfkA): | GGAACAATCACCCAAAAATAAGAGAAACTTCGTGGTGGCTACGACTT |
| *pfkA*-down-F: | TTCTCTTATTTTTGGGTGATTGTTCC |
| *pfkA*-down-R: | cggtacccggggatcctctagAGAACGGTGGTACGAACATCG |
| *gap*-up-F: | aacgacggccagtgccaagctACTGGTTGGATGTGCTCGAAG |
| *gap*-up-R: | CACAGATTCAAATTAATGTCAACCCTAGGTCAATCAGAGCATCGGTCAC |
| P*_eftu_*-F(gap): | GGGTTGACATTAATTTGAATCTGTGCACAGGGTAGCTGGTAGTTTG |
| P*_eftu_*-R(gap): | GGAAATGCAATGTGTCAAGCAGGAAACTTCGTGGTGGCTACGACTT |
| *gap*-down-F: | TTCCTGCTTGACACATTGCATTTCC |
| *gap*-down-R: | cggtacccggggatcctctagGTGGAAACCAGTGGCTCTTCG |
| *pyk*-up-F: | aacgacggccagtgccaagctCATCGGTGGACGCATTTATC |
| *pyk*-up-R: | CACAGATTCAAATTAATGTCAACCCAGACAATGTTCTACGCCTCTG |
| *P_eftu_*-F(pyk): | GGGTTGACATTAATTTGAATCTGTGCACAGGGTAGCTGGTAGTTTG |
| *P_eftu_*-R(pyk): | GATGAATCTTGCGACCCAGAGGAAACTTCGTGGTGGCTACGACTT |
| *pyk*-down-F: | TTCCTCTGGGTCGCAAGATTCATC |
| *pyk*-down-R: | cggtacccggggatcctctagGGATCTTTGCCCACTGAAGTT |
| *pgi*-up-F: | cggtacccggggatcctctagTGCTTGGCTGGTAGAGAAGCT |
| *pgi*-up-R: | GCGTAATCACGTTGGCTCTTTTCTGCTGCCCTATTTGCGCGGTACCACTT |
| *pgi*-down-F: | GAAAAGAGCCAACGTGATTACGCAGCAGCTGTTTAGTCGCTTGCTTATAGGATCAG |
| *pgi*-down-R: | aacgacggccagtgccaagctCACCATTACCGATGAGAAACATG |
| *zwf*-up-F: | cggtacccggggatcctctagCATCGACGACGTTCGCAAT |
| *zwf*-up-R: | GTCTCCTTCCTTCTGCCTAGGCTCAACGGCCACATAAGATCGAAC |
| *zwf*-down-F: | GAGCCTAGGCAGAAGGAAGGAGACACGACATGAGCACAAACACGACCCCCT |
| *zwf*-down-R: | aacgacggccagtgccaagctGCCAATATCTTCAGCCATGGT |
| *tkt*-up-F: | cggtacccggggatcctctagTGTCCAAGGGTTCTCCTCCAG |
| *tkt*-up-R(Ptac): | AACACAGATTCAAATTAATGTCAACCGGTTCGTTTTCGTTAGATCCG |
| *tkt*-down-F(Ptac): | GGTTGACATTAATTTGAATCTGTGTTATAATGGTTCAACTTTGGTCCCGGTTTAACC |
| *tkt*-down-R: | aacgacggccagtgccaagctATGAAGGTAGGTCGCTTGGTG |
| *cg3035*-RT-F: | GCATTGCGGAGCGTTCTAATT |
| *cg3035*-RT-R: | ATCGGCGTCAATGACTTTCTG |
| *cat*-RT-F: | CAGTGCCAATCAACAAGCCAG |
| *cat*-RT-R: | TTCGCTCTCCAGGAAGTCGAG |
| *pta*-RT-F: | AGCGCCGAACTCTTTGAAAAC |
| *pta*-RT-R: | AGCGCGCTCCTTGATCTTTAC |
| *ack*-RT-F: | GGCCGCATCGTACTCAAAAT |
| *ack*-RT-R: | ACTGCGGTGATTTCCAGTTG |
| *tkt*-RT-F: | ACAACCGCATCTCCATCGAAG |
| *tkt*-RT-R: | GAACGCGGATGAAGGTAGGTC |
| *zwf*-RT-F: | CAACACTCAAGCGCATCGACA |
| *zwf*-RT-R: | TTGAGCTCGTGTGCGGATTCG |

Restriction enzyme sites were represented as bold characters. The overlapping region with PCR fragment were marked by underline. The overlapping region with vector was represented as lowercase.

Additional file 1: Table S2. Promoter and terminator sequence used in this study

| Name | sequence (5’-3’) |
| --- | --- |
| *P_tac_* | TTGACATTAATTTGAATCTGTGTTATAATGGTTC |
| *P_sod_* | AGCTGCCAATTATTCCGGGCTTGTGACCCGCTACCCGATAAATAGGTC  GGCTGAAAAATTTCGTTGCAATATCAACAAAAAGGCCTATCATTGGGA  GGTGTCGCACCAAGTACTTTTGCGAAGCGCCATCTGACGGATTTTCAAA  AGATGTATATGCTCGGTGCGGAAA |
| *P_eftu_* | CACAGGGTAGCTGGTAGTTTGAAAATCAACGCCGTTGCCCTTAGGATT  CAGTAACTGGCACATTTTGTAATGCGCTAGATCTGTGTGCTCAGTCTTC  CAGGCTGCTTATCACAGTGAAAGCAAAACCAATTCGTGGCTGCGAAAG  TCGTAGCCACCACGAAGT |
| T | GGGCGGTCAGATGATCGCCCTTTTTTTTTT |
| RBS(*zwf*) | GAGCCTAGGCAGAAGGAAGGAGACACGAC |


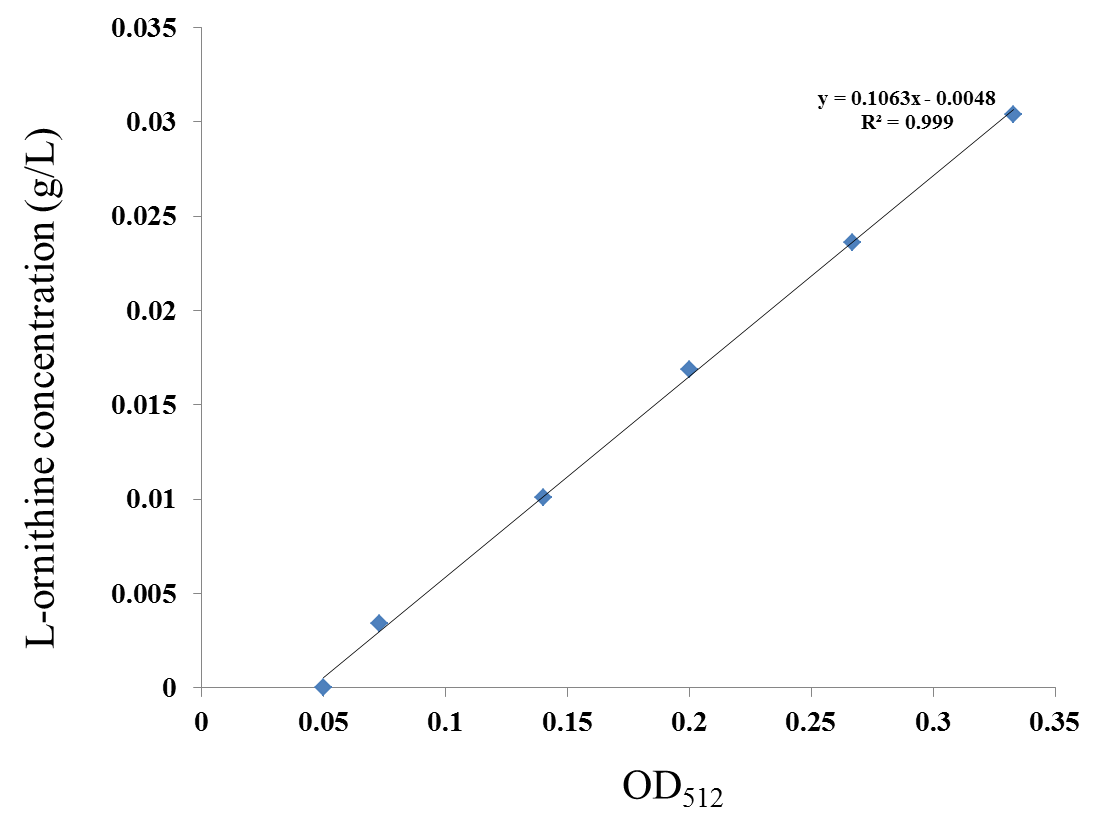


Additional file 1: Figure S1. l-Ornithine standard curve of colorimetric assay using ninhydrin
